# Review of Israel’s action and response during the COVID-19 pandemic and tabletop exercise for the evaluation of readiness and resilience—lessons learned 2020–2021

**DOI:** 10.3389/fpubh.2023.1308267

**Published:** 2024-01-24

**Authors:** Khitam Muhsen, Dani Cohen, Aharona Glatman-Freedman, Sari Husseini, Saritte Perlman, Carrie McNeil

**Affiliations:** ^1^Department of Epidemiology and Preventive Medicine, School of Public Health, Faculty of Medicine, Tel Aviv University, Tel Aviv, Israel; ^2^Middle East Consortium on Infectious Disease Surveillance, Jerusalem, Israel; ^3^Israel Center for Disease Control, Israel Ministry of Health, Ramat Gan, Israel; ^4^Ending Pandemics, San Francisco, CA, United States

**Keywords:** readiness, resilience, COVID-19, Israel, during action review, tabletop exercise

## Abstract

**Background:**

Reevaluating response plans is essential to ensuring consistent readiness and resilience to the COVID-19 pandemic. The “During Action Review” and Tabletop (DART) methodology provides a retrospective and prospective assessment to inform the adaptive response. Israel introduced COVID-19 vaccinations in December 2020 and was the first country to implement booster vaccination to address waning immunity and surges caused by new variants. We assessed Israel’s readiness and resilience related to COVID-19 response while capturing the pre-vaccination and vaccination periods.

**Methods:**

A DART analysis was conducted between December 2020 and August 2021 among experts involved in the management of the COVID-19 pandemic in Israel. During the retrospective stage, a role-based questionnaire and discussions were undertaken in a participant-led review of the response, focusing on epidemiology and surveillance, risk communication, and vaccines. The prospective stage included tabletop exercises to evaluate short to long-term simulated scenarios.

**Results:**

Participants emphasized the pivotal role of Israel globally by sharing experiences with the pandemic, and vaccination. Perceived strengths included multi-sectoral collaboration between the Ministry of Health, healthcare providers, academia, military, and others, stretching capacities, expanding laboratory workload, and establishing/maintaining surveillance. The vaccine prioritization plan and strong infrastructure, including computerized databases, enabled real-life assessment of vaccine uptake and impact. Challenges included the need to change case definitions early on and insufficient staffing. Quarantine of patients and contacts was particularly challenging among underprivileged communities. Risk communication approaches need to focus more on creating norms in behavior. Trust issues and limited cooperation were noted, especially among ethnic and religious minorities. To ensure readiness and resiliency, participants recommended establishing a nationally deployed system for bringing in and acting upon feedback from the field, especially concerning risk communication and vaccines.

**Conclusion:**

Our study appraised strengths and weaknesses of the COVID-19 pandemic response in Israel and led to concrete recommendations for adjusting responses and future similar events. An efficient response comprised multi-sectoral collaboration, policy design, infrastructure, care delivery, and mitigation measures, including vaccines, while risk communication, trust issues, and limited cooperation with minority groups were perceived as areas for action and intervention.

## 1 Introduction

The COVID-19 pandemic caused a substantial global burden, including over 770 million cases and 6.95 million deaths by August 2023 (1). Mitigation measures in the first year, before COVID-19 vaccines became available, relied on non-pharmaceutical interventions, including lockdowns, contact tracing and isolation, canceling mass gatherings, and imposing wearing of face masks in public spaces, which varied in effectiveness (2–7). These measures were swiftly enforced and frequently altered, which might have affected the public trust in policymakers, resilience, and compliance with these measures (8–11).

When the first wave of the COVID-19 pandemic struck, strengths and gaps in readiness became quickly apparent. Therefore, understanding the impact of COVID-19 on health and societies, especially in the early phases before the vaccination era, is crucial to reflect on the lessons learned and anticipate potential future scenarios.

Tools such as the World Health Organization (WHO)‘s Intra-Action Reviews retrospectively assess response capabilities during a response (12). To understand how well prepared a country will be for future health emergency scenarios, tabletop exercises (TTXs) bring response organizations together to prospectively identify strengths and gaps in readiness (13). Knowledge learned through After Action reviews of real-life and TTXs have been used to update planning documents and improve response to COVID-19—even as events are unfolding (14–17). There are advantages of conducting evaluations using participatory methodologies, mainly ensuring that those directly involved in response lead in its evaluation and in identifying appropriate solutions to benefit the community involved (18, 19).

In Israel, COVID-19 preparedness began before the WHO declared a pandemic; by the end of January 2020, a national emergency was declared (20). In February–March 2020 restrictions on international travel and mass gatherings were enforced, and community transmission of SARS-CoV-2 was established later, in March 2020. The first national lockdown was imposed between March 17 and April 19, 2020, resulting in “converting the curve” and lifting the restrictions thereafter (20). However, a marked surge led to a second lockdown during September–October 2020 (20). Israel was among the first countries to introduce COVID-19 vaccination in December 2020 using the BNT162b2 mRNA vaccine, in parallel to a lockdown implemented between December 27, 2020, and February 7, 2021 (20–22). The COVID-19 vaccination campaign in Israel led to rapid and high vaccine uptake, which was highly effective in preventing COVID-19-related hospitalizations and deaths (23, 24). However, in the early stages of the COVID-19 vaccination campaign, uptake was lower among residents of low versus high socioeconomic status communities and among the Arab and ultraorthodox Jewish populations compared to the general Jewish population (20, 21). The aim of the current study was to implement the During Action Review and Tabletop (DART) participatory approach for evaluating Israel’s COVID-19 surveillance response, vaccine deployment capabilities, and risk communication. The rationale for focusing on these topics was that they cover essential public health responses.

## 2 Methods

### 2.1 Study population and design

Israel is a high-income country among OECD members (25, 26). As of the end of 2020, Israel’s population comprised of 9.28 million people; 74% Jewish, 21% Israeli Arabs, and ~ 5% of other ethnicities (27). About 12% of the total population belongs to the ultraorthodox Jewish (religious) population. These groups differed by the COVID-19 incidence and mortality rates, COVID-19 vaccine uptake, and SARS-CoV-2 testing (20).

The Ministry of Health (MOH) is the main regulator of the healthcare system in Israel. A universal healthcare insurance law has been implemented since 1995 (28), which provides all citizens with a “basket” of universal health services including primary prevention, immunizations, outpatient and inpatient services through four Health Maintenance Organizations (HMOs), and high access to healthcare (28).

The management of the COVID-19 pandemic in Israel was led by the MOH, involving cross-ministry teams and collaboration across healthcare providers, government, civil society, academic organizations, and the private sector. It addressed all the complexities related to containment of the virus transmission, treatment of COVID-19 patients, expanding testing capacities, enforcement of regulations, and restrictions, purchasing medical equipment and vaccines, establishing and maintaining the infrastructure needed for mass vaccination, electronic systems to enable real-life evaluation of the infection spread and vaccine uptake and impact, and more (29). The existing National Emergency Authority was not activated during the COVID-19 pandemic, rather the management of the pandemic was led by the MOH, which had Coronavirus czar, who was responsible on national and sub-national policy, in addition to *ad-hoc* and well-established consultants/working groups/teams on all aspects of the pandemic such as the Epidemic Management Team (EMT) and the National Immunization Technical Advisory Group (NITAG). The founding of the “Alon Headquarters” in August 2020 is an example of cross-discipline collaboration between MOH and the Home Front Command of the Israel Defense Force (IDF). The “Alon Headquarters” assisted in conducting the epidemiological investigations and contact tracing, as well as digital surveillance for contact tracing, and enforcement of self-isolation. Moreover, the Ministry of Defense assisted in purchasing medical equipment in the early phases of the pandemic and supported communities under lockdown, while elite intelligence bodies provided complementary expertise (29, 30). The national emergency medical, disaster, ambulance and blood bank service (Magen David Adom—MDA), also played a major role in supporting the pandemic management, such as establishing SARS-CoV-2 testing facilities, conducting testing at home for quarantined individuals and vaccinating the population (29, 31–34). The cross-sector collaborations between governmental and academic institutions generated high-quality research regarding Israeli’s experience with COVID-19 pandemic which assisted other countries in decision-making (35).

### 2.2 Data sources

We used publicly available anonymized aggregate data on COVID-19 in Israel, including SARS-CoV-2 testing, number of cases, hospitalizations, and related deaths, as well as COVID-19 vaccine uptake, to describe the context of the study.

We also utilized reports obtained in the framework of a DART exercise, as previously described (36), conducted during the second and third waves of the pandemic, capturing both the pre-vaccination and the early vaccination eras in Israel. The DART assessment was conducted among experts in public health and health professionals who were directly involved in the management of the pandemic.

### 2.3 DART approach

A five-step DART approach was utilized to allow for participant-led prospective and retrospective evaluation (36). The key features of DART methodology are that it is a flexible and modular approach, follows one health approach where applicable, it is co-developed with in-country leadership, it is scenario-based and participants-led assessment (36). In the DART assessment of Israel, experts opted to focus on surveillance, risk communication, and vaccines, which are limited to human health, but still, we took a multisector approach. Clinical care response and maintenance of essential health services were not covered in this DART evaluation.

#### 2.3.1 Step 1: the development of a “during action” review questionnaire and discussion

We developed an initial open-ended questionnaire to gather information from participants in critical response roles, using the modular DART questionnaire templates related to the following roles: Epidemiology, Surveillance, and Communications. Questions were modified to address specific issues, concerns, and operations in Israel. We approached experts in public health, health professionals, and researchers who took a role in the efforts of COVID-19 pandemic management and invited them to participate in the DART assessment. Those who agreed were asked to fill in a role-based questionnaire. Completed questionnaires were analyzed and comprised the basis for a facilitated, two-hour, participant-led discussion to identify priority strengths and gaps in the response up to that date. During the discussion, participants also noted the most concerning future scenarios for how the pandemic may unfold; these findings were used to inform the TTX design (Step 2).

#### 2.3.2 Step 2: designing the “tabletop exercise” (TTX)

Design began with a *Concept and Objectives* meeting in which objectives for the TTX were developed using data collected in Step 1 regarding critical gaps in response capabilities and future scenarios of concern. Based on input from Step 1, it was determined that this TTX focuses on three response roles: Epidemiology and Surveillance, Behavior, and Risk Communication, as well as Vaccines and Mitigation Measures. Role-based scenarios were designed to include the following concerns identified in Step 1: future waves with ineffective vaccines due to new strain emergence and transmission, vaccine hesitancy, new pandemics or concurrent major outbreaks, natural disasters, political situations, long-term impacts of social isolation, recession impacting supply availabilities and compliance with non-pharmaceutical and personal protection measures. These scenarios also reviewed capability needs identified during Step 1, including logistics and coordination, adaptive management, addressing health disparities/concerns of minority communities, and community wellbeing and compliance.

The TTX incorporated multiple role-based scenarios in “phases” looking at different advance time-frames: one near-term scenario happening three months later, September 2021, and another scenario occurring in December 2023. Between each phase, participants answered assessment questions: “What did you feel most prepared for?,” “What did you feel least prepared for?,” “What actions could be taken today to strengthen your ability to respond to this scenario?” and “Did the scenario highlight or bring to mind any other potential scenarios to plan for in the future?”

#### 2.3.3 Step 3: conducting TTX using START_X_

The multiplayer, multi-scenario TTXs were conducted remotely online in June 2021 using the Scenario-based Tool for Assessing Readiness through Tabletop Exercises (START_x_) (37). TTXs were designed to be completed asynchronously over one week to allow for those actively engaged in a response to complete when available to do so. Participants were asked to answer questions as they would during the actual response, based on current plans and protocols. The TTX was conducted in Hebrew and included two scenarios to assess readiness for challenges that might occur in the pandemic in September 2021 and December 2023 (Boxes 1, 2).

BOX 1 **Main points of Scenario 1—September 2021.**

- All social restrictions lifted, increase in travel and international visitors.
- Vaccine uptake leveled off at 61% with high rates of non-masking indoors, with concerns rising about increasing re-infection among those infected last year.
- Vaccine hesitancy is rising in adults; low rates of parents plan to vaccinate their children.
- New, more infectious strain has led to an increase in severe breakthrough cases among the vaccinated. Children appear to be more likely to become ill with the new strain.
- Vaccine rates have also stayed low within a few minority communities; social media reports that COVID-19 restrictions have been enforced more heavily in these communities, and now refuse to cooperate with epidemiological investigations for fear that the information will be used to generate further charges against them.
- Wildfires pose a threat to case identification and response.

BOX 2 **Main points of Scenario 2—December 2023.**

- COVID-19 has become endemic, moving around the population much like the common cold.
- SARS-CoV-2 has shown a substantial ability to mutate, leading to numerous known variants globally, many of those strains have been confirmed to be circulating in Israel.
- Annual booster shots for COVID-19 have become common, although rates of vaccination with boosters have been lower than with the original COVID-19 vaccine. Because there is no international agreement on which variants should be included, the contents of each year’s booster vary by manufacturer and country. 5% of cases remain serious among the general population; 3% among those fully vaccinated still result in hospitalization and/or death.
- Long COVID has led to years-long disabilities including difficulty concentrating, shortness of breath, and fatigue leading to many having to leave the workforce.
- A severe influenza season is already underway in much of the world, and flu rates have been picking up dramatically in Israel. Low influenza rates in previous years caused the influenza vaccination rate to drop below 20%. Vaccination campaigns are underway but have been hampered by a sense of complacency and a mistaken belief that the COVID-19 booster also provides protection against influenza.
- A new variant has emerged that is both more transmissible and pathogenic with risk of severity and death much more than that of the original SARS-CoV2 strain; even higher among those with comorbid influenza.
- Existing vaccines are only 20% effective against this variant, but manufacturers are developing a new vaccine for it, tentatively scheduled for distribution beginning in February of 2024.
- Due to the great success of the previous COVID-19 vaccine efforts, health officials are confident in their ability to quickly obtain a high vaccination rate.
- Other countries have already implemented a return to pandemic-style lockdowns, leading to loud outcries among the Israeli public against returning to lockdown. Some local leaders have responded by pledging that they would refuse to shut schools or businesses again. Images of burning medical masks have become common in people’s feeds as a symbol against a return to lockdown. Among certain communities, there are active misinformation campaigns suggesting that the government is exaggerating the severity of the new strain to have an excuse for controlling the movements and activities of the population.
- Heavy rains, flash-flooding pose impacts to effective response.

#### 2.3.4 Step 4: TTX after action review

Once the TTX was completed, a draft of the TTX ‘After Action Review’ (AAR) report downloaded from START_x_ was sent to participants to share each role-based scenario and responses from each participant. Sharing the AAR allowed all participants to have a common operating picture since they were each playing pieces of the scenario unique to their roles. During the AAR discussion, participants identified overarching needs and recommendations for strengthening capabilities to improve future resiliency and readiness.

#### 2.3.5 Step 5: final and comprehensive assessment report

Participant findings were collated into a comprehensive assessment report.

### 2.4 Ethics approval

Only anonymized aggregate data were used in this study, which was approved by the Ethics Committee of Tel Aviv University.

## 3 Results

**Figure 1:**
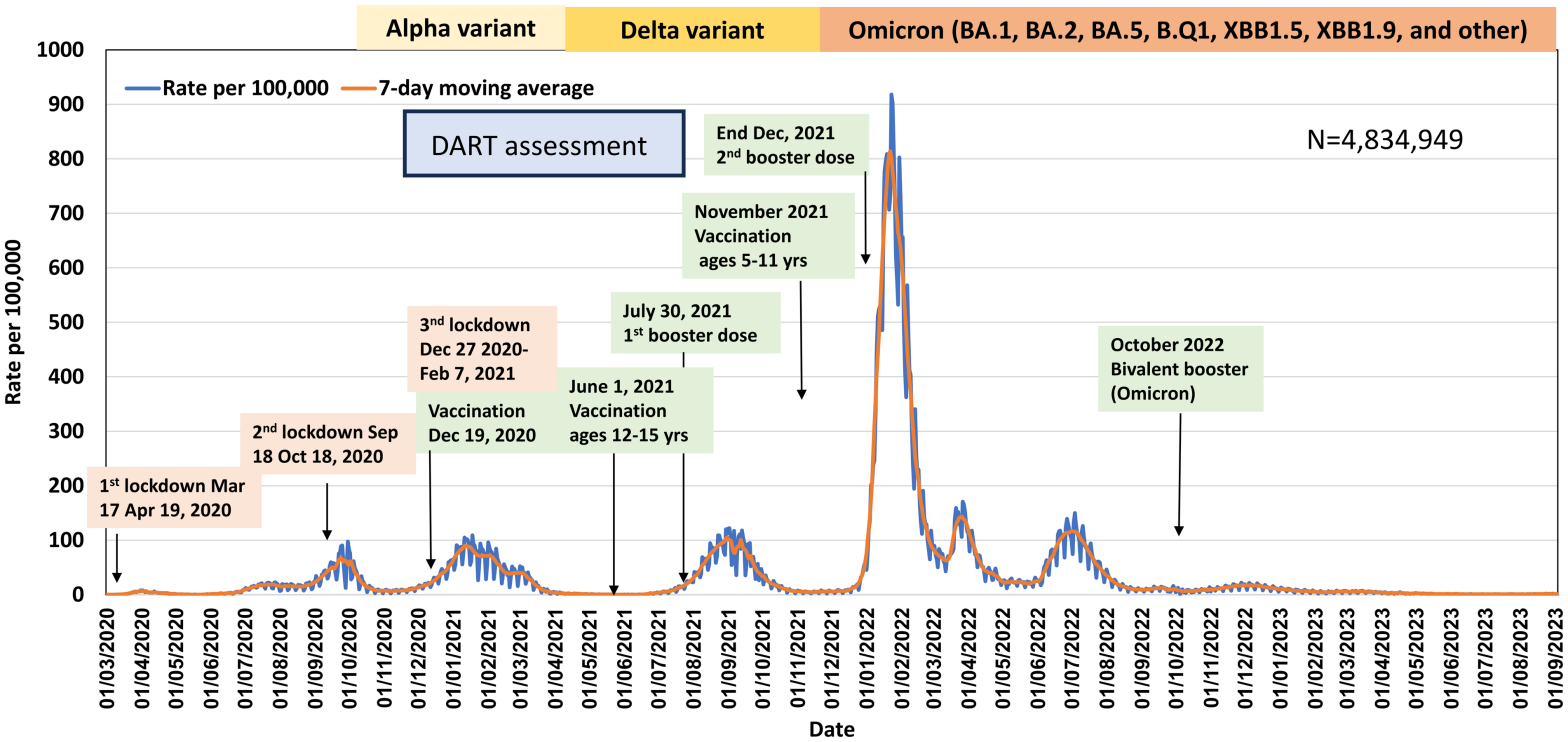
Daily incidence rates of overall SARS-COV-2 infection, Israel, March 2020–September-2023.

**Figure 2:**
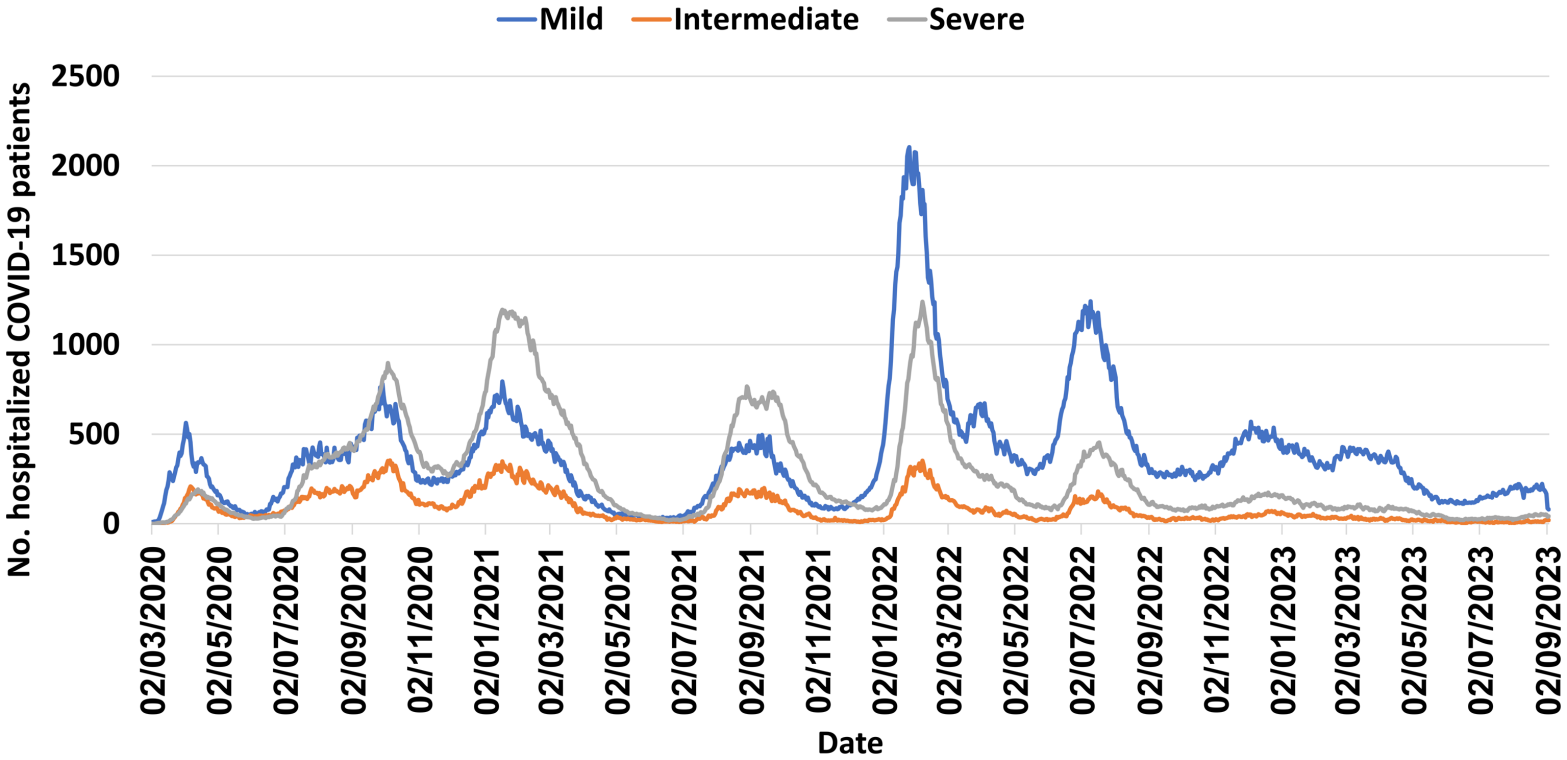
Number of hospitalized COVID-19 patients in Israel, March 2020–September 2023.

**Figure 3:**
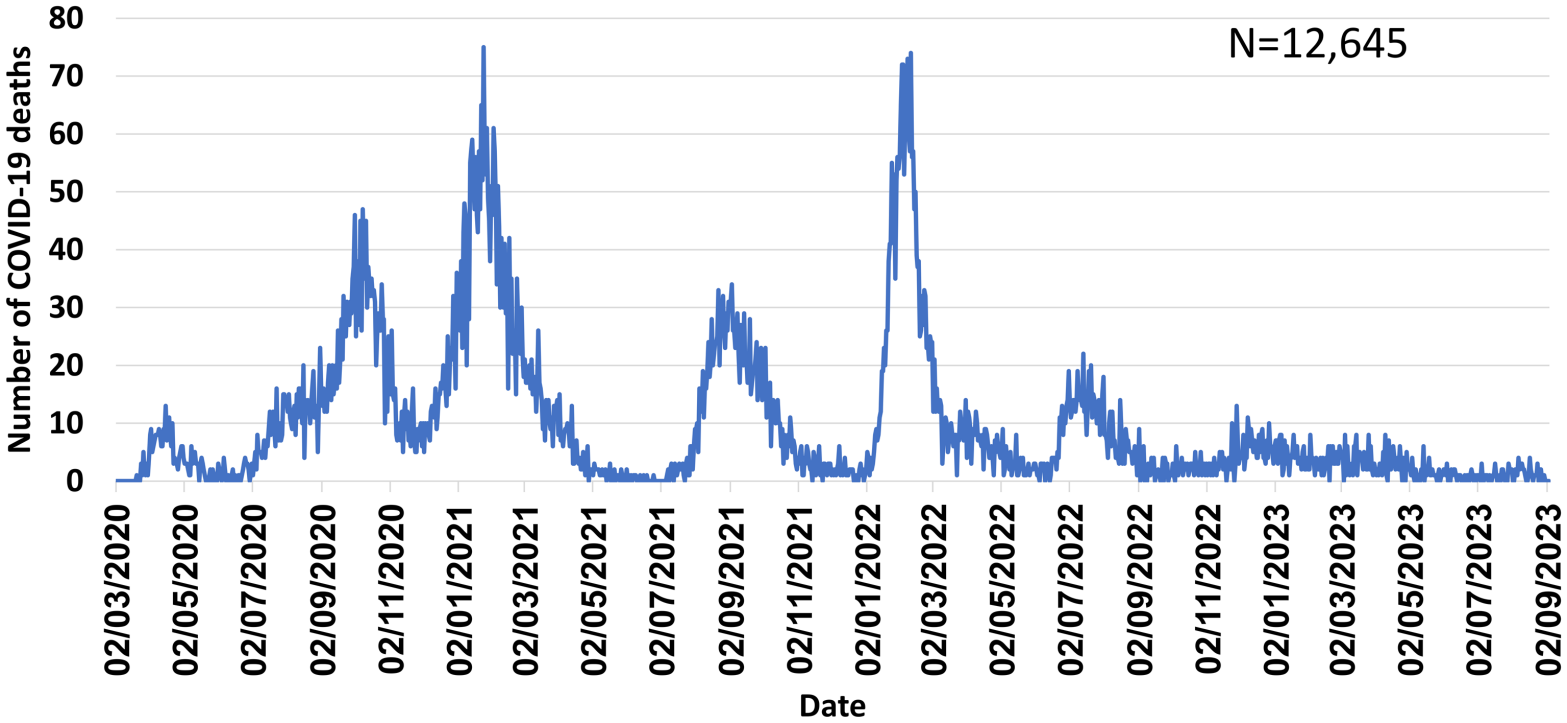
Number of COVID-19 deaths in Israel-March 2020–September 2023.

**Figure 4:**
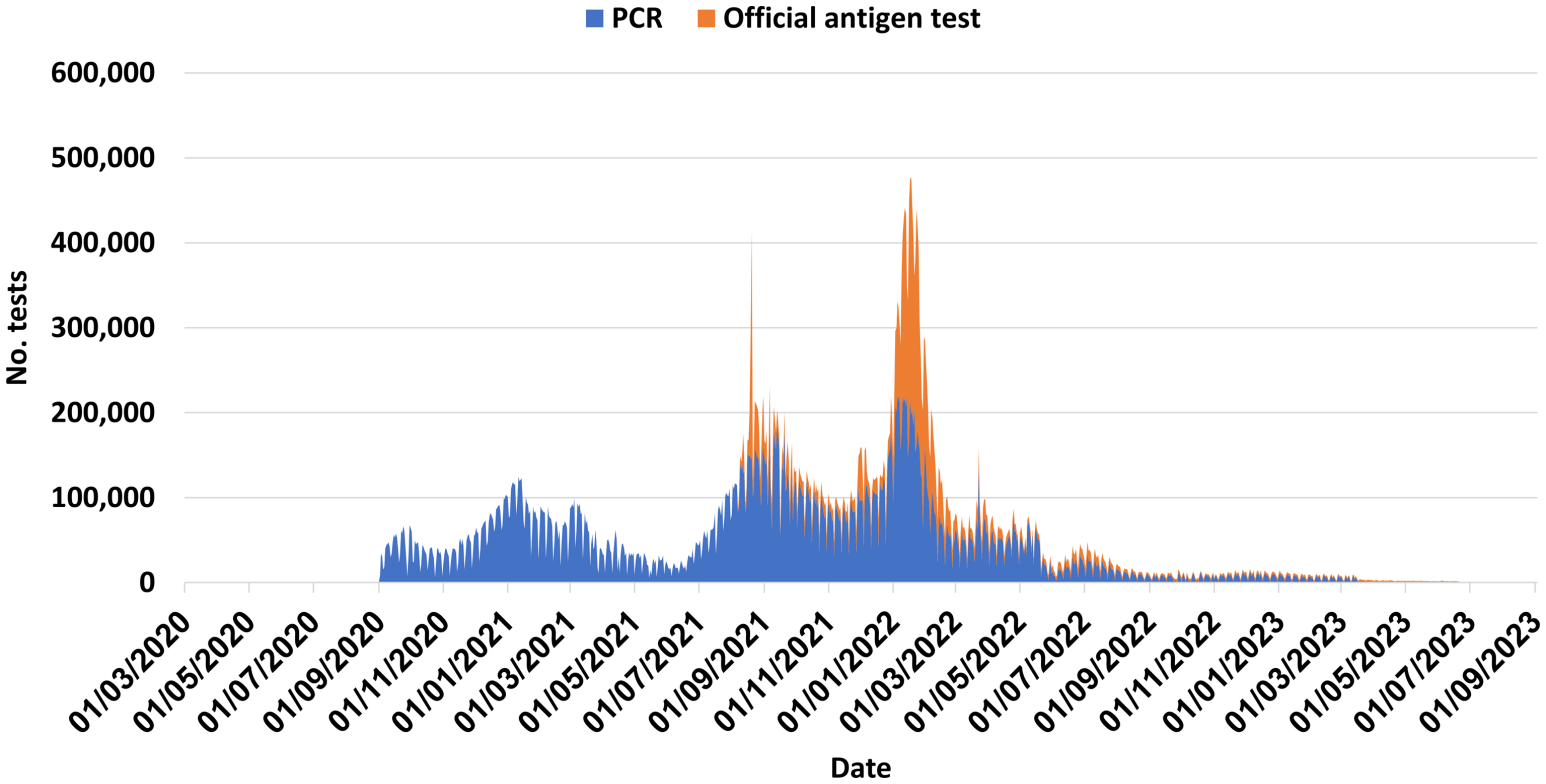
Number of daily SARS-CoV-2 tests, March 2020-Septmber 2023, Israel.

Like other countries, multiple COVID-19 waves occurred in Israel, caused by various variants, highest incidence observed during the Omicron wave (Figures 1–3). Figure 4 shows the expansion of COVID-19 testing during various waves. In the first year of the pandemic, three lockdowns were imposed in Israel, a policy that was replaced by booster vaccination to mitigate disease surges caused by the Delta and Omicron variants in the second year of the pandemic (Figure 1).

### 3.1 The “during action” review questionnaire and the tabletop exercise

The DART assessment was undertaken from December 2020 to August 2021, in which eight experts participated. Their field of expertise included public health, epidemiology, infectious diseases, vaccinology, risk communication, and nursing.

#### 3.1.1 Strengths in readiness and resiliency

DART demonstrated numerous strengths in the Israeli response.

##### 3.1.1.1 Vaccination campaign

At the time of the retrospective during action review, it was noted that the vaccine campaign had made tremendous progress since the questionnaire (vaccine coverage was approximately 25%, the most of any country in the world at that time). Participants highlighted the leadership role Israel has played globally in sharing data related to COVID-19 mitigation and response, particularly the vaccination campaigns.

Participants noted vaccine distribution was based on an effective prioritization plan formulated by an expert committee. The universal health care also aided the prioritization and identification of target group; healthcare is not considered a privilege but rather a right in the country. In addition, the existing and newly added systems also contributed to success in implementing the vaccination schedule by the HMOs and other organizations. Healthcare workers, among the first receiving the vaccine, served as role models for the rest of the population. Vaccine adverse effects were tracked through a well-developed digital communication system. Different vaccine manufacturers were approached to expand access to vaccines as needed.

##### 3.1.1.2 Communication

An initial effective risk communication by MOH was noted as a key success in the response to the vaccination campaign. Transparency and availability of information were perceived as beneficial. The risk communication strategy required a comprehensive approach. Effective coverage included multiple methods such as television advertising, radio, and online outlets, including social media. Social media messaging has focused on using humor to engage younger audiences—which was highlighted as both a strength and an area to improve.

##### 3.1.1.3 Epidemiology and surveillance

With all Israelis covered by a national registry that was created to manage the epidemic, the MOH had the digital abilities to allow for effective surveillance in the response. Surveillance data were reported daily and shared with the public and media. The fact that they have been able to identify new variants showed the strength of detection within the surveillance system, and laboratory capacities.

Additional strengths included: adapting existing system for tracking other infectious diseases to tracking COVID-19, trace back and investigation (epidemiological investigation findings informed operational decision-making to increase the protection of healthcare workers and contact tracing within 24–48 h), case definitions based on international guidelines, routine screening for healthcare workers in internal medicine and geriatric wards and institutions [e.g., ‘Senior Shield’ operation (31, 32, 38)], mitigation measures (quarantine for exposed and suspected cases, masks required in public places, recommendations of avoidance of public gatherings, vaccine purchase agreements with multiple manufacturers) and transferring logistic responsibilities to the IDF Home Front Command to break chain of infection.

Regarding community support and resiliency, while capacities have been stretched, the participants highlighted bringing in the military, local pharmacists, and others to assist in epidemiologic investigations. By working with universities, private laboratories, and other facilities beyond classical public health laboratories, the response increased testing capabilities markedly. Additionally, strong commitment from the public health and medical communities, openness to the adoption of new technologies, and increasing lab capacity and resilience of the population accustomed to emergencies, were perceived as strengths by the various experts.

The tabletop exercise highlighted the role Israel played in sharing data with the world to inform response, especially regarding their early and efficient vaccine administration.

#### 3.1.2 Challenges in the response to date

**Figure 5:**
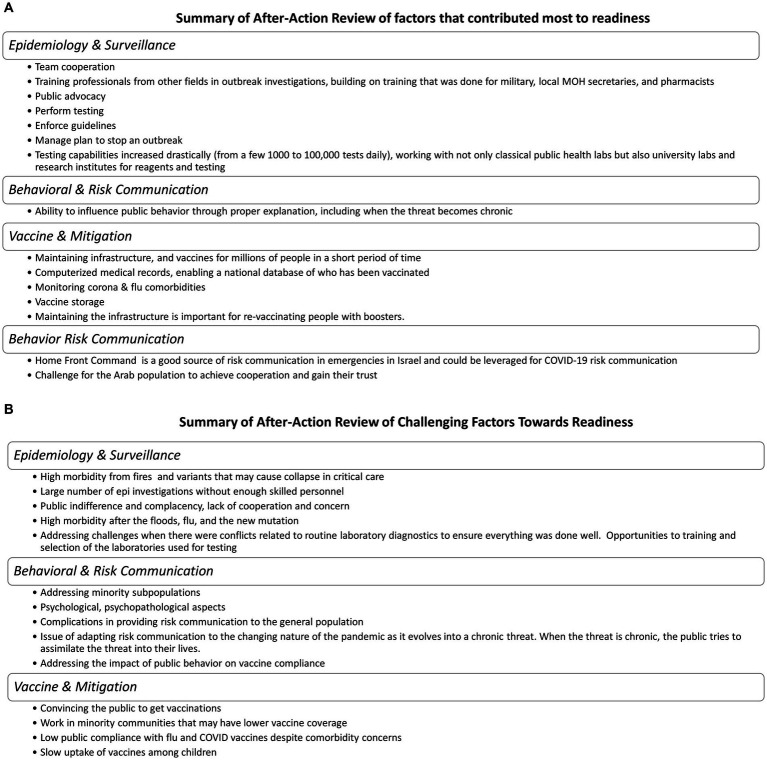
**(A)** Summary of after-action review of factors that contributed most of readiness. **(B)** Summary of after-action review of challenging factors toward readiness.

During the initial phases of the pandemic, Israel faced the challenges of surveillance, with changing case and exposure definitions, and symptoms. Isolation for large families and among the poor made control difficult, compounded by the risks of asymptomatic infection. The response was hindered at times by a lack of sufficient staffing; however, as noted above, the military and others were trained and brought in to address needs from logistics to epidemiology. COVID-19 burden on understaffed hospitals may have left other non-COVID patients neglected. Future scenarios demonstrated a need to develop a plan to address surge needs when either a vaccine-resistant variant overwhelms capacity or when floods, fires, or concurrent outbreaks stress the health and public health systems. The TTX portion highlighted the lack of sufficient epidemiology and critical care capacity during high morbidity concurrent incidents such as fires, influenza, floods, or new variants (Figure 5).

Initial risk communication had differing reviews among participants; one noted that a fear-based approach led to public distrust and cynicism. As the pandemic shifts from acute to chronic, participants advised that risk communication approaches need to focus on helping create norms in behavior. Including military risk communication experts in future messaging development was also recommended. After working through future scenarios in the TTX, participants expressed concern regarding how to best address public indifference and complacency, particularly in the later months and years of the pandemic. The scenarios also demonstrated challenges in reaching minorities and in building the trust needed for effective risk communication.

Throughout the pandemic and in the future scenarios projected, participants noted trust issues and lack of cooperation make it hard to track cases and vaccinate minority groups. Language barriers further complicated outreach to these communities.

Political interference in professional decision-making was noted as an obstacle to effective response at times. Economic impacts and pandemic fatigue lowered the resiliency of the general population to the ongoing surges (8).

### 3.2 Recommendations

#### 3.2.1 Priority action items

Priority Action Items focused on what participants called the “root solutions”: vaccination and behavior. These included (1) vaccinating those who are not vaccinated, providing booster vaccination to those over 60 years of age, and incorporating family physicians to stop new disease waves, and (2) including training on response and vaccines in medical schools and continuing education for physicians to improve communication with the public (3) reverting to a “traffic light” or staged plan, such as was used early in the pandemic, which would be informed by public health professional guidance.

#### 3.2.2 Policy

Participants noted that it would be critical to ensure that decision-makers understand that preparedness and behavioral change require a deep understanding of how people understand signs of risks and how they respond to them. To address this, participants recommended more rapid processing of data and trends for the purpose of making optimal policy modifications. Participants also stated it is important that the position and role of the public health professionals be maintained to inform policy and empower the standing of professional recommendations to reduce conflict between politicians and public health professionals.

#### 3.2.3 Plans

In terms of preparedness planning, participants recommended that response plans be developed consisting of several stages, which would be determined according to incidence data and guidance by public health professionals—such as the “stop light” models. Participants also recommended that implementation plans focus on public cooperation regarding enforcing guidelines to reduce morbidity.

#### 3.2.4 Protocols

The review found that during times of high incidence during the pre-vaccination era, school closures and switching to remote learning would be effective, along with limitations on gatherings, encouraging mask usage, and limiting flights from countries with high levels of new variants. Pre-alignment with pharmaceutical companies for the option of additional mass purchases of vaccines with a quick supply turnaround to prevent shortage, was also recommended.

#### 3.2.5 Addressing public indifference, complacency, noncompliance, misinformation

Communication emerged as a critical area of need in this assessment. Recommendations included having professionals communicate messages to the public, developing a national plan for handling false information, and establishing nationally deployed mechanisms to receive feedback from the field. Timing of communication was identified as a key factor. Specifically, participants advised that establishing communication with the public occur as soon as possible to explain the need for vaccines. As physicians were perceived to be trusted sources, improving their knowledge of vaccinology and vaccines was highlighted as a recommendation. Participants identified a need to focus on norms and normative behavior rather than continually changing laws and guidelines, or developing sanctions.

The assessment highlighted how communication and trust building was a unique challenge when working with minority subpopulations. Recommendations to address this gap included ensuring that professionals, including physicians, were well-trained about vaccines and risk communication, engaging community leaders, and utilizing culturally tailored communication. Participants also recommended preparing and evaluating the efficacy of advocacy in increasing vaccination among subpopulations.

### 3.3 Data sharing to strengthen regional and global resilience

The global crises caused by the COVID-19 pandemic highlighted the importance of harmonized and collaborative work by disease surveillance networks within and across countries. Data sharing as well as sharing of experiences and mitigation measures was pivotal to strengthening both regional and global resilience and readiness. Accordingly, the platform of existing regional and global networks was utilized to achieve this goal. The Middle East Consortium for Infectious Disease Surveillance (MECIDS), a non-governmental organization comprising leading public health officials and academics from Israel, Jordan, and the Palestinian Authority, provided a trusted platform to enhance regional collaboration when facing the COVID-19 pandemic, while considering the needs of all partners. MECIDS played a significant regional role in the exchange of knowledge and data sharing of COVID-19 surveillance and laboratory detection methods among public health experts from Jordan, the Palestinian Authority, and Israel. Knowledge and data exchange included providing professional updates on the status of the pandemic status in each country, exchange of experience related to COVID-19 vaccination, training health professionals in COVID-19 related epidemiology and laboratory aspects, education of the public regarding SARS-CoV-2 and its transmission as well as preventive measures including vaccines. At the global level, the experience accumulated by MECIDS from its significant regional engagement was shared with similar CORDS networks (Connecting Organizations for Regional Disease Surveillance, a network of networks) coordinated by Ending Pandemics (United States), across East and West Africa, Europe and East and South Asia. CORDS network activities included monthly joint meetings and discussions as well as regular webinars which served as important means of exchanging in-depth experiences acquired during the pandemic.

## 4 Discussion

We described the main COVID-19 pandemic control measures implemented in Israel, and using the DART approach, we assessed Israel’s readiness and resiliency between December 2020 and August 2021, capturing the pre-vaccination era, early vaccination period and readiness for potential future complications that COVID-19 may pose.

Our study focused on public health aspects, mainly epidemiology/surveillance, vaccines, and risk communication. Notably, experts emphasized the pivotal role of Israel globally by sharing experiences related to COVID-19 vaccination impact and effectiveness, as reflected by numerous scientific publications from Israel (20–24, 31, 32, 35, 38, 39), as well as the intensive meetings, webinars and training conducted jointly with regional and global partners such as MECIDS and CORDS. Experts’ perceived strengths of Israel’s COVID-19 response included multi-sectoral collaboration between the MOH, healthcare providers, academia, and other organizations, stretching capacities, expanding laboratory workload, establishing/maintaining surveillance, designing and implementing the vaccine prioritization plan. The experts further mentioned the strong infrastructure, including electronic health records, that resulted in a successful vaccination campaign, rapid vaccine deployment, high uptake and rapid impact on morbidity and mortality (21–24, 40, 41). These elements were shown to be important in preparedness for other emerging infectious diseases, such as the 2009 H1N1 pandemic (42, 43). The WHO also identified these elements as major areas for action to strengthen health systems, as demonstrated in the “six-building blocks” framework: service delivery; health workforce; information; medical products, vaccines, and technologies; financing; and leadership and governance (44). Haldane et al. (45), in their assessment of health systems in managing the COVID-19 pandemic in 28 countries, identified four elements of resilience that characterized highly effective country responses, including the activation of comprehensive responses, adapting capacity within and beyond the health system to address the needs of communities; preserving functions and resources within and beyond the health system to maintain care delivery of services, and lessening vulnerability (45). The experts’ perceived strengths of Israel’s response to the pandemic fall within these elements. Our findings were also confirmed in a literature review demonstrating that surveillance, risk and vulnerability assessments, prediction and decision-making, alerts, and early warnings are critical components of epidemic detection and early warnings, as well as control and preparedness-preventive strategies (46).

Israel’s response to the COVID-19 pandemic in the early phases was rapid and successful as reflected by relatively low mortality and incidence rates, and “averting the curve.” This is likely attributed to the implementation of multiple interventions, including various limitations on international travel imposed early during 2020, extensive contact tracing and self-isolation (quarantine) program, enhances surveillance, canceling mass gatherings, school closures and strict lockdowns (20, 47, 48). These activities enabled the healthcare system, time to prepare for the treatment of COVID-19 patients (47). Israel was among the first countries the introduce the COVID-19 vaccines once they became available in December 2020 following a well-defined prioritization plan. The implementation of the COVID-19 vaccination program was also successful, resulting in high vaccine uptake and effectiveness. The high access to vaccines, well-developed healthcare and logistic infrastructure, coupled with strong collaboration between the MOH and healthcare providers, good advertising campaign, likely contributed to the success of Israel’s response during the vaccination period (21, 22).

The experts in our study noted that expanding surveillance and laboratory capacities was a main strength that enabled the detection of new variants of concern. This is in line with the WHO efforts to enhance the use of genomic surveillance as a pandemic preparedness and response tool. Indeed, in many countries, SARS-CoV-2 genomic sequencing capability increased markedly within a short period (49). Eventually, these efforts led to the WHO’s establishment of The Global Genomic Surveillance Strategy for Pathogens with Pandemic and Epidemic Potential 2022–2032 in March 2022 to provide greater coherence to support genomic surveillance (49).

Other resilience and readiness evaluations focused on hospitals’ capacities to deliver care (50), the resilience of healthcare workers (51) or building information technology systems in hospitals (52). These domains and those capitalized in our study are vital for a comprehensive country-level readiness assessment. Interestingly, the cross-sectoral government-academic collaboration, integrating academic research in outbreak response, was also highlighted as a major strength in other settings (53).

The main challenges that were noted by the experts in Israel’s COVID-19 response included frequent case definition changes early on, insufficient human resources, especially healthcare workers, limited cooperation by some ethnic and religious minorities, difficulty enforcing quarantine for patients and contacts in underprivileged communities, weaknesses in risk communication approaches and trust issues in policy makers. These findings reflect vulnerability in adaptability to control measures enacted by public health authorities and posed pressure on various sectors of the healthcare system, and differentially affected certain population groups during the pandemic. A study conducted among US residents of the Gulf South who experienced the COVID-19 pandemic alongside climate-related disasters showed that individuals who spoke English as their primary language, had higher education and higher levels of resilience, were found to have a significantly better pandemic preparedness, which also correlated with disaster preparedness (54). A study from Belgium explored information needs, coping mechanisms with COVID-19 mitigation measures, and their effect among racialized/ethnic minority communities (55). Findings from this indicated a need for tailored and timely information and that an insufficiency of official public health messages uncovered a negative impact of mitigation measures on citizen’s livelihoods as well as a distrust in authorities (55). Community-based initiatives blunted this impact using culturally tailored intervention and outreach activities (55). These shared findings suggest a pivotal role of social determinants in the COVID-19 pandemic. Therefore, integrating social sciences in epidemic preparedness and response is likely warranted (56), to strengthen individual and community levels of resilience.

Vulnerability and resilience represent two related complementary approaches describing systems and actors’ responses to change and shock (57). Notably, the DART methodology captured well both aspects in Israel’s early response to the COVID-19 pandemic. As such, DART methodology can be useful either as a stand-alone tool or a complimentary tool for the assessment of health systems’ response to the pandemic and future emergencies, such as the Health System Response Monitor (HSRM), which was established by the WHO European Regional Office and the European Commission (58). The HSRM analysis also demonstrated a range of health system challenges and weaknesses across Europe, showing that countries prioritized policies on investing in public health, supporting the workforce, maintaining financial stability, and strengthening governance in their response to the COVID-19 pandemic (58).

DART’s approach allowed the experts to make recommendations that were designed to ensure continued improvement in readiness and resiliency. The main recommendations were expanding COVID-19 vaccination, including booster vaccination and engagement of family physicians to mitigate potential new waves. Participants also recommended establishing a nationally deployed system for bringing in and acting upon feedback from the field, especially regarding risk communication and vaccines. These recommendations were communicated with the MOH and stakeholders involved in the response to the COVID-19 pandemic and were mostly considered in adapting the country response. The continued partnership between the MOH and the academia, characterized by mutual interests of enhancing resilience and control of the pandemic and, is important to address future public health emergencies.

Our study has several strengths. DART allowed retrospective and prospective assessment of Israel’s readiness and resilience to cope with COVID-19. In-depth insights were gained from multiple role actors who had served in various roles in the management of the pandemic in Israel. We followed a flexible approach in both selecting the main themes for evaluation and providing sufficient time for the experts to complete the various aspects of the assessment. Collectively this resulted in a real-life reliable, and comprehensive evaluation and recommendations.

Our study has several limitations. Our DART assessment covered mainly the first year of the pandemic, thus, it might not fully capture all the strengths and limitations of Israel’s response to the pandemic. Nonetheless, our analysis captured the main periods of the pre-vaccination era, the first vaccination campaign, and touched the booster vaccination periods, thus ensuring a lengthy assessment of various aspects of the response. Moreover, the future scenarios exercise was well-designed and predicted well the emergence of new variants of concerns and events that could be stressors to mitigation efforts of the pandemic. DART assessment required the participation of experts who played a role in managing the pandemic. This was challenging since experts were busy with day-to-day management activities. To address this concern, DART allowed flexibility and provided sufficient time to complete all elements and generate a comprehensive assessment and recommendations.

In summary, the DART assessment demonstrated the strengths of Israel’s COVID-19 resilience and preparedness response and identified gaps that should be strengthened in future emergency events. An efficient response was characterized by being multidisciplinary, including multi-sectoral collaboration, policy design, infrastructure, care delivery, and mitigation measures, including vaccines, while risk communication, trust issues, and limited cooperation with minority groups were perceived as areas for action and intervention. Enhancement of regional resilience activities and global partnerships should be maintained.

## Data availability statement

The datasets presented in this study can be found in online repositories. The names of the repository/repositories and accession number(s) can be found at: https://datadashboard.health.gov.il/portal/dashboard/corona.

## Ethics statement

The studies involving humans were approved by Tel Aviv University, Tel Aviv, Israel. The studies were conducted in accordance with the local legislation and institutional requirements. The ethics committee/institutional review board waived the requirement of written informed consent for participation from the participants or the participants’ legal guardians/next of kin because Using anonymized aggregate data.

## Author contributions

KM: Conceptualization, Data curation, Formal analysis, Funding acquisition, Investigation, Methodology, Supervision, Writing – original draft. DC: Conceptualization, Investigation, Supervision, Writing – review & editing, Funding acquisition, Methodology. AG-F: Investigation, Writing – review & editing. SH: Investigation, Project administration, Writing – review & editing. SP: Data curation, Investigation, Project administration, Software, Writing – review & editing. CM: Conceptualization, Formal analysis, Funding acquisition, Investigation, Methodology, Project administration, Resources, Software, Supervision, Writing – original draft.
